# A flexible ancestral genome reconstruction method based on gapped adjacencies

**DOI:** 10.1186/1471-2105-13-S19-S4

**Published:** 2012-12-19

**Authors:** Yves Gagnon, Mathieu Blanchette, Nadia El-Mabrouk

**Affiliations:** 1 Département d'Informatique (DIRO), Université de Montréal, H3C 3J7, Canada; 2 McGill Centre for Bioinformatics, McGill University, H3C 2B4, Canada

## Abstract

**Background:**

The "small phylogeny" problem consists in inferring ancestral genomes associated with each internal node of a phylogenetic tree of a set of extant species. Existing methods can be grouped into two main categories: the distance-based methods aiming at minimizing a total branch length, and the synteny-based (or mapping) methods that first predict a collection of relations between ancestral markers in term of "synteny", and then assemble this collection into a set of Contiguous Ancestral Regions (CARs). The predicted CARs are likely to be more reliable as they are more directly deduced from observed conservations in extant species. However the challenge is to end up with a completely assembled genome.

**Results:**

We develop a new synteny-based method that is flexible enough to handle a model of evolution involving whole genome duplication events, in addition to rearrangements, gene insertions, and losses. Ancestral relationships between markers are defined in term of *Gapped Adjacencies*, i.e. pairs of markers separated by up to a given number of markers. It improves on a previous restricted to direct adjacencies, which revealed a high accuracy for adjacency prediction, but with the drawback of being overly conservative, i.e. of generating a large number of CARs. Applying our algorithm on various simulated data sets reveals good performance as we usually end up with a completely assembled genome, while keeping a low error rate.

**Availability:**

All source code is available at http://www.iro.umontreal.ca/~mabrouk.

## Background

One of the aims of comparative genomics is to reveal the evolutionary scenario that has led to an observed set of present-day genomes from hypothetical common ancestors. When a speciation history, represented as a phylogenetic tree, is already known, the problem reduces to that of finding ancestral genomes, in terms of gene content and organization, for non-terminal nodes of the tree. The reconstruction of ancestral karyotypes and gene (or any other type of markers) content and order has been widely considered by the computational biology community [1-7]. For most formulations in terms of different kinds of genomes (circular, multichromosomal, single or multiple gene copies, signed or unsigned genes) and different distance metrics, even the simplest restriction in term of the median of three genomes, has been shown NP-hard [8]. As reviewed in [9,10], the considered methods can be grouped into two main classes. The distance-based methods aim at labeling ancestral nodes in a way minimizing total branch length over the phylogeny [3,6,7,9]. On the other hand, the synteny-based (or mapping) methods [2,4,5,11] rely on three steps: (1) Infer a collection of ancestral genes; (2) Infer a collection of relations between ancestral genes in terms of "synteny"; (3) Assemble this collection into an ancestral genome. In contrast to a distance-based approach, the output of a synteny-based approach is a set of Contiguous Ancestral regions (CARs) that is not guaranteed to be completely assembled into a genome. However, the predicted CARs are likely to be more reliable as they are more directly deduced from observed conserved features of the extant species.

The first formal method based on this approach was developed by Ma *et al*. [5]. In this algorithm, syntenies are adjacencies, sets of ancestral relations are computed by a variant of the Fitch parsimony algorithm and a greedy heuristic is used for the assembly. Another class of synteny-based methods [4,12] define ancestral relations in term of common intervals, represent them in a binary matrix, and then solve a problem known as the *Consecutive Ones problem (C1P) *to translate the matrix into sets of ancestral CARs. When the collection of ancestral relations is fully compatible, the translation into ancestral genomes is straightforward, but in general the problem of transforming the matrix into a C1P matrix in an "optimal" way is hard, and appropriate simplifications are considered. The result of such methods is not a unique ancestral gene order but rather a PQ-tree representing a collection of possible orders.

Most computational methods for comparative genomics account only for markers with exactly one copy in every considered extant genome. A few extensions to genomes with unequal gene content have also been considered [2,12,13]. The case of multiple gene copies is more challenging as the one-to-one correspondence between orthologs is missing. Recently, a number of ancestral genome inference studies have accounted for multiple gene copies in the very special case of an evolution by Whole Genome Duplication (WGD). WGD is a spectacular evolutionary event that has the effect of simultaneously doubling all the chromosomes of a genome. Evidence of WGD has shown up across the whole eukaryote domain. A distance-based approach for inferring a pre-duplicated genome was developed in 2003 [14], and extended to the median problem [15-17]. However, the synteny-based approach is more naturally extendable to WGD events. Indeed, as the pre-duplicated genome has single gene copies, as long as an appropriate way for inferring "Double Conserved Synteny" (DCS) relations between ancestral markers is found, the assembly part can be taken without any modification. In [18], Gordon *et al*. manually reconstructed the ancestral yeast genome. Formal extensions of the synteny-based approach to handle WGD have also been developed [2,9,19]. In this paper, we present a new synteny-based method for ancestral genome inference, allowing for evolutionary scenarios involving WGDs and gene losses, where relations between ancestral genes are defined as *Gapped Adjacencies*, i.e. pairs of genes separated by up to a fixed number of genes. It is an extension of a previous method [2] where relations between genes were defined in term of "direct" adjacencies. The assembling step is based on the computation of a rigorous score for each potential ancestral gapped adjacency (*g, h*), reflecting the maximum number of times the gapped adjacency between *g *and *h *can be conserved along the branches of the whole phylogeny, over any possible setting of ancestral genomes. To make the link with the C1P framework, the syntenies that we consider in this paper can be related to gapped gene teams, while those considered in [4] are related to various types of common intervals [20]. However the assembly methods and the output of the algorithms (a set of CARs versus a PQ-tree) are very different. In the absence of WGD events and gene losses, the approach most comparable to ours is the one developed by *Ma et al*. [5]. In case of direct adjacencies, the algorithm in [2] obtained a higher accuracy for adjacency prediction than *Ma*'s algorithm, but at the cost of a higher number of CARs, preventing from recovering a completely assembled genome. In this paper, relaxing the constraint of adjacency to gapped adjacency allows to improve on these results. Indeed, our results on simulated data sets show that we usually end up with a completely assembled genome, while keeping a low error rate.

## Methods

### Problem statement and preliminary concepts

The general problem we are aiming to solve can be stated as follows.

**Input: **A set Γ of *m *modern genomes, a species tree *S *with leaves labeled with genomes from Γ, and an internal node *ν *of *S *representing a speciation event of interest;

**Output: **An ancestral genome at node *ν*.

Formally, a species tree (or phylogeny) for Γ is a tree *S *with *m *leaves, where each genome of Γ is the label of exactly one leaf, and each internal node (called *speciation node*) has exactly two children and represents a speciation event. We say that *S *is *labeled *if each internal node *u *of *S *has a label *G*(*u*) corresponding to a hypothetical ancestral genome just preceding the considered speciation event.

**Figure 1:**
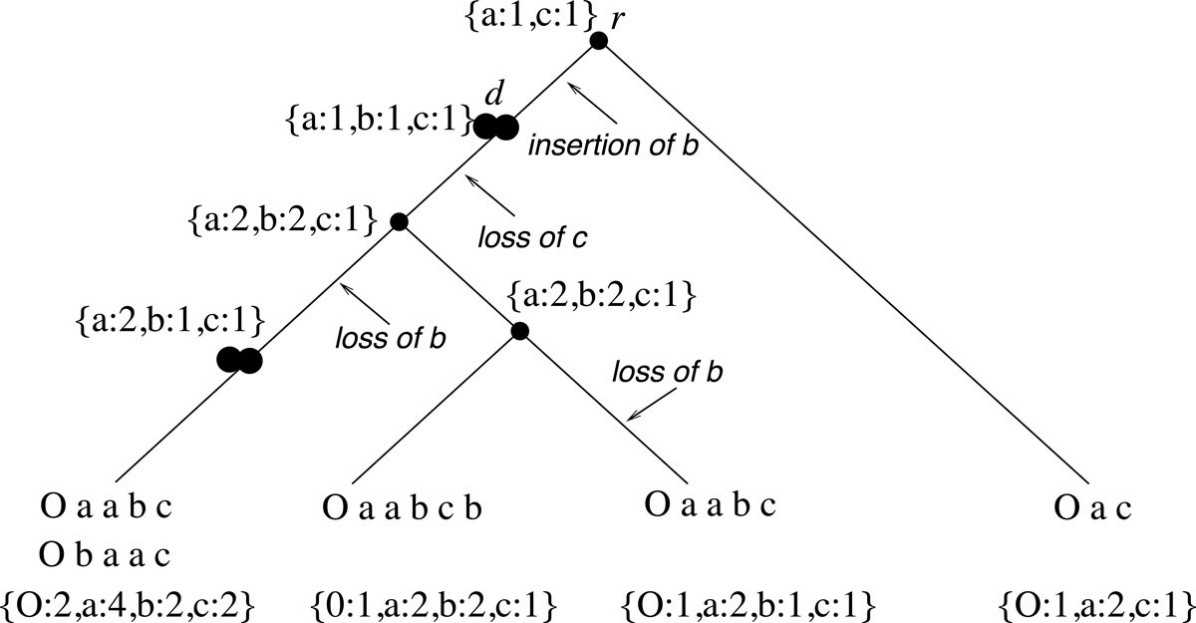
**A species tree with each leaf labeled with its corresponding genome**. For simplicity, we consider all the genes to be positively signed. The last line below each leaf is the gene set and multiplicity of each gene. Single circles indicate speciation nodes, while double-circles indicate WGD nodes. Applying the procedure described in the text leads to the gene set assignment and multiplicity given as labels of internal nodes. This assignment leads to the indicated insertion and losses.

Considering a set Σ of genes, a genome is a set {*C*_1_, *C*_2_, ... *C_N_*} of chromosomes, where each chromosome is a sequence of signed elements from . Chromosomes can be circular or linear, but we always use a circular representation by adding an artificial gene *O *at the end of a linear chromosome and considering the augmented chromosomes as circular. Given a genome *G*, we call the gene set of *G *and denote by $\sum_{G}\subseteq \sum$ the set of genes present in *G *(including *O*). For example, the gene set of the genome labeling the leftmost leaf of the tree in Figure 1 is {*O, a, b, c*}. We further denote by $\pm \sum_{G}$ the set obtained from $\sum_{G}$ by considering each gene in its positive and negative directions. By convention, the gene *O *is always considered positive. A *multiset *of $\pm \sum_{G}$ is a subset of $\pm \sum_{G}$ with possibly repeated genes. Given a gene $g\in \sum_{G}$, we denote by *mult*(*g, G*) the *multiplicity*, i.e. number of copies, of ±*g *in *G*. In particular, the multiplicity of *O *is the number of linear chromosomes of *G*. For example, the multiplicity of gene *a *in the genome of the leftmost leaf of the tree in Figure 1 is 4. We extend our notation to define, for node *u *of the tree, $\sum_{u}$ and *mult*(*g, u*) as the set of genes present in the genome at node *u *and the multiplicity of *g *in that genome.

#### Evolutionary model

Our model involves rearrangements and content-modifying operations. As we adopt a synteny-based approach, rearrangements are only implicitly considered, as only traces of these rearrangements in terms of disrupted gene adjacencies are considered. In other words, all kinds of rearrangement events can be present in the history. Our approach also allows for unequal gene content, resulting from gene losses or insertions. As for the multiplicity of genes, the only operation leading to multiple gene copies (genes with multiplicity ≥ 2) that is considered is the *Whole Genome Duplication *(WGD). Formally, a WGD is an event transforming a genome *G *= {*C*_1_*,C*_2 _*... C_N_*} of *N *chromosomes into a genome *G*^D ^containing 2*N *chromosomes, i.e. $G^{D}=\left\{C_{1},C_{1}^{\prime },C_{2},C_{2}^{\prime }...C_{N},C_{N}^{\prime }\right\}$, where, for each $1\leq i\leq N,C_{i}=C_{i}^{\prime }$.

In addition to the assumption that WGDs are the only events responsible for gene multiplicity (in particular, single-gene duplications are not considered), we suppose that, in each genome, at least one gene reflects the doubling status of the genome, i.e. there exists a gene that has not lost any copy. As noticed by Zheng *et al*. [17], under these assumptions, the number and position of WGD events can be easily deduced from the multiplicity of the most frequent gene found in each genome. To account for such events, new internal nodes, called *WGD nodes*, are added appropriately on the edges of *S *(see Figure 1). As for speciation nodes, a label *G*(*u*) of a WGD node *u *represents an ancestral genome just preceding the WGD event (i.e. containing a single copy of each gene). Contrary to speciation nodes, each WGD node has only a single child. Moreover, if all extant genomes have a gene with multiplicity greater than 1, then a WGD node is inserted above the root of *S*.

#### Adjacencies

Each gene $g\in \sum_{G}$ is represented by an ordered pair of unsigned markers, its start and end, with +*g *represented as (*g^t^, g^h^*) and -*g *represented as (*g^h^, g^t^*). Genome *G *can thus be thought of as a sequence of pairs of markers. We say that a gene $b\in +\sum_{G}\left(\text{respectively}-b\in -\sum_{G}\right)$ is a left *α*-adjacency of a gene $a\in \pm \sum_{G}$ in *G *if both genes are on the same chromosome and the number of markers between *b^h ^*(respectively *b^t^*) and *a^t ^*in *G *is smaller than *α*. Symmetrically, $b\in +\sum_{G}\left(\text{respectively}-b\in -\sum_{G}\right)$ is a right *α*-adjacency of $a\in \pm \sum_{G}$if the number of markers between *a^h ^*and *b^t ^*(respectively *b^h^*) is smaller than *α*. In other words, when *α *is odd, *b *is left *α*-adjacent to *a *iff *G *contains substrings *b × a *or -*a x *- *b*, where *x *is a sequence of at most (*α *- 1)/2 signed genes from $\pm \sum_{G}-\left\{a,b\right\}$. When *α *is even, *b *is left *α*-adjacent to a iff (i) *b *is (*α *- 1)-adjacent to *a *or (ii) *G *contains substrings -*b x a *or -*a x b*, where *x *is a sequence of at most (*α *- 2)/2 signed genes from $\pm \sum_{G}-\left\{a,b\right\}$. For $g\in \sum_{G}$, we use *LA*(*g, α, G*) (resp. *RA*(*g, α, G*)) to denote the *multiset *of signed genes that are left (resp. right) *α*-adjacent to *g*. For example, for the genome G labeling the leftmost leaf in the tree of Figure 1, we have *LA*(*a*, 1,*G*)= {*O, a, b, a*}, while *RA*(*a*, 2, *G*) = {*a, b, a, c*, -*a*, -*b*, -*a*, -*c*}.

#### Conserved adjacencies

Consider a branch (*u, v*) of a labeled tree *S*, where *v *is a descendent of *u*. To assess the level of conservation between an ancestral gene arrangement *G*(*u*) and its descendant *G*(*v*), we compare the left and right *α*-adjacency multisets in *G*(*u*) and *G*(*v*). More precisely, we define adjCons(g,α,G(u),G(v))=|LA(g,α,G(u))∩LA(g,α,G(v))|+|RA(g,α,G(u))∩RA(g,α,G(v))|, as the number of left and right conserved *α*-adjacencies of *g *on (*u, v*), and $adjCons\left(\alpha ,G\left(u\right),G\left(v\right)\right)=\underset{g\in \Sigma_{u}\cap \Sigma_{v}}{\sum }\;adjCons\left(g,\alpha ,G\left(u\right),G\left(v\right)\right)$ as the number of conserved *α*-adjacencies on the branch (*u, v*). Finally, the number of conserved *α*-adjacencies over the whole tree *S*, denoted as *adjCons*(*α, S*), is the sum of *adjCons*(*α, G*(*u*),*G*(*v*)) for all branches (*u, v*) of *S *Notice that in *adjCons*(*α, G*(*u*),*G*(*v*)) we account for each adjacency conservation twice. It may appear that right adjacencies alone (or, symmetrically, left adjacencies) are sufficient to reflect adjacency conservation between two genomes. But consider, for example, the sequence "+1 -2 +3 -4". If we just consider right 1-adjacencies, then the subsequence "+ 1 -2" will be considered twice (as -2 is the right adjacency of 1 and -1 is the right adjacency of 2) but the subsequence "-2 + 3" will not be considered (as -3 is the left adjacency of 2 and -2 is the left adjacency of 3).

### Ancestral gene content

The first step of our ancestral inference method is to assign ancestral gene content and multiplicity at each ancestral node. Resolving the ordering of these genes is performed in a second step. We consider a natural procedure, inspired from [18], assuming a model with no convergent evolution and minimum losses. We say that a node *v *is a *direct descendant *of a WGD node *u *if and only if *v *is a WGD node or a leaf and there is no other WGD node on the branch from *u *to *v*. To assign gene content Σ_*u *_and gene multiplicity at each internal node *u *of *S*, we apply the two following operations in two bottom-up traversals of *S*: (1) For each WGD node *u *and each gene *g*, let *v *be the direct descendant of *u *with maximum multiplicity for *g*. If *mult*(*g, v*) ≥ 2 then assign *g *to *u *and define $mult\left(g,u\right)=\left\lfloor \frac{mult\left(g,v\right)}{2}\right\rfloor$. For example after a traversal of the species tree *S *of Figure 1, the gene set of the WGD node *d *only contains *a *and *b*, as the maximum multiplicity of *c *in the direct descendants of *d *is 1; **(2) **Assign a gene *g *to any internal node *u *of *S *on a path from the node of *S *representing the least common ancestor (LCA) of all the nodes containing *g *(leaves or WGD nodes), to any leaf containing *g*. Moreover, if not already defined, define *mult*(*g, u*) as the maximum multiplicity of *g *in *u*'s children.

In the rest of this paper, we will assume that gene content and multiplicity is set for all nodes of *S*. A *labeling G*(*u*) of a node *u *of *S *will refer to a genome respecting the content and multiplicity constraints given by Σ_*u*_. Notice that, by construction (taking the maximum multiplicity of each gene at each internal node), there is no increase of multiplicity (except possibly from 0 to 1 in the case of the gain of a new gene) from a node *u *to a child *v*, unless *u *is a WGD node, in which case the multiplicity of a gene is at most doubled. Such a construction guarantees that any labeling of *S *can be explained by an evolutionary scenario in agreement with the hypothesis of WGDs being the only events responsible for gene multiplicity.

### A synteny-based method accounting for direct adjacencies

In [2], we have presented a synteny-based method that infers a pre-duplicated ancestral genome at a node *ν *corresponding to a highest WGD node of *S*, or any node preceding a first WGD node. More precisely, the method aims at infering a genome *G*(*ν*) such that *adjCons*(1,*S*|*G*(*ν*)) is maximized, where *adjCons*(1, *S*|*G*(*ν*)) is *adjCons*(1,*S*) (as defined in Section Conserved Adjacencies, for *α *= 1) with the constraint that genome *G*(*ν*) is assigned at node *ν *(see details in [2]).

For any node *u *of *S *and any gene *g *of $\sum_{u}$, let *X *be a multiset of *mult*(*g, u*) potential adjacencies of *g*. Define *LeftAdj*(*g, S*|_*LA*(*g*,1,*G*(*u*)) = *X*_) (resp. *RightAdj*(*g, S*|_*RA*(*g*,1,*G*(*u*))=X_)) as the maximum number of left (resp. right) adjacencies that can be preserved over the whole tree, for any ancestral genome assignment with the constraint that the genome *G*(*u*) satisfies *LA*(*g*, 1,*G*(*u*)) = *X*. The following upper bound on the objective function allows to treat each gene independently:

$$adjCons\left(1,S|G\left(u\right)\right)\leq \sum_{g}LeftAdj\left(g,S{|}_{LA\left(g,1,G\left(u\right)\right)=X}\right)+RightAdj\left(g,S{|}_{RA\left(g,1,G\left(u\right)\right)=X}\right)$$

The method, that we call DirectAdj, proceeds in two steps summarized below.

**Figure 2:**
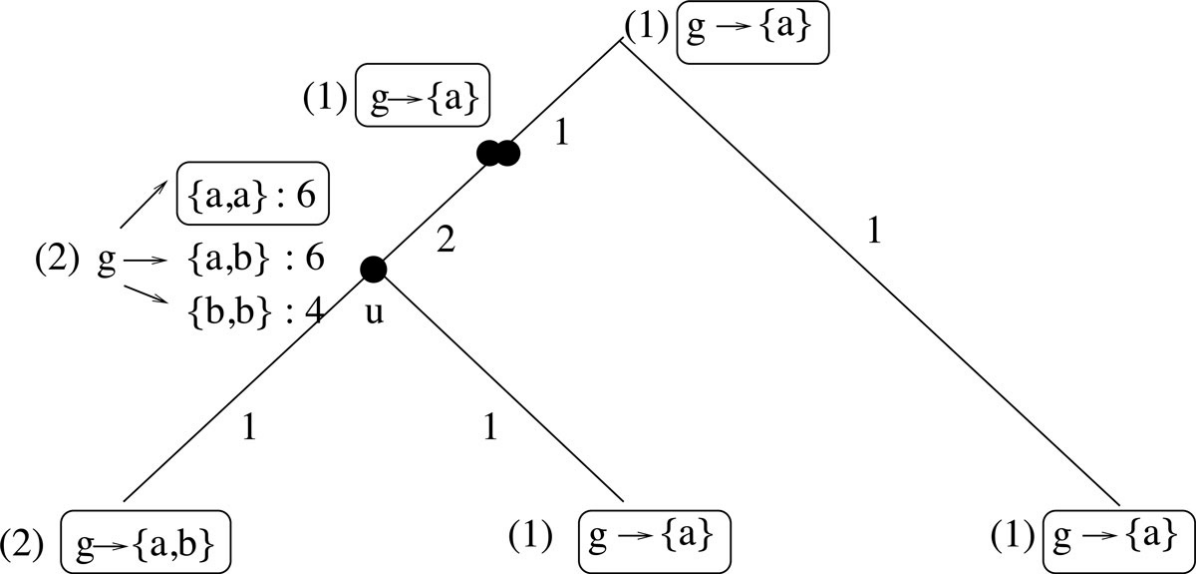
**An illustration of Step 1 for a gene *g *and an internal node *u***. Numbers in brackets indicate the multiplicity of gene *g *at each node of the tree. Multisets at leaves represent (say left) adjacencies of gene *g *in the corresponding genome. All multisets *X *of possible adjacencies of *g *at node *u *are shown, followed by the value of *LeftAdj*(*g, S*|_*LA*(*g*,1,*G*(*u*))=X_). The rest of notation illustrates how the value 6 is obtained at *u *for the multiset {*a, a*}: the root and WGD node labels are the adjacencies that have to be set for *g*, and the label of an edge (*v, w*) is the number of conserved adjacencies for *g *on that branch.

**Step 1**: For each internal node *u *of the tree (speciation or WGD node), each gene $g\in \sum_{u}$, and each multiset *X *of possible adjacencies of *g *at node *u*, we compute *LeftAdj*(*g, S*|_*LA*(*g*,1,*G*(*u*)) = *X*_) and *RightAdj*(*g, S*|_*LA*(*g*,1,*G*(*u*)) = *X*_) using a Dynamic Programming Algorithm. The values at a node *u *are computed from the values at the two children and also at the parent of *u *(see Figure 2 for an example).

**Step 2**: For the node *ν *for which an ancestral genome is sought, we obtain the desired pre-duplicated genome by chaining adjacencies. As *ν *represents a speciation preceding any WGD event, or a first WGD event, each gene *g *of $\sum_{v}$ is present exactly once at *ν*. At this node we use the notations $L\left(g,h\right)=LeftAdj\left(g,S{|}_{LA\left(g,1,G\left(v\right)\right)=\left\{h\right\}}\right)$ and $R\left(g,h\right)=RightAdj\left(g,S{|}_{RA\left(g,1,G\left(\nu \right)\right)=\left\{h\right\}}\right)$. We proceed by a reduction to the Traveling Salesman Problem (TSP) on a complete undirected graph *Q *where vertices correspond to genes, and an edge (*g, h*) is weighted according to a ratio (*L*(*g, h*) + *R*(*h, g*))/*MaxAdj*(*g, S*), where *MaxAdj*(*g, S*) is the number of nodes of *S *containing *g*. The division by *MaxAdj*(*g, S*) allows to correct for genes that are lost in some parts of the tree, which avoids favoring genes with high multiplicity. Moreover, as noticed in [2], the result of the TSP is usually a single long chromosome concatenating all genes. To avoid this drawback, we define TSP-*τ *by augmenting the initial TSP heuristic with the procedure of cutting, from the inferred ancestor, all adjacencies with weight less than a certain threshold *τ *(see Algorithm section). All details on costs, the heuristic used to solve the TSP and how to handle chromosomal endpoints and gene signs, are given in [2]. The value of *τ *has been chosen to optimize accuracy on simulated datasets, according to the error rate associated to the set of adjacencies of a given weight. However, a conservative threshold in term of error rate leads to an excess of CARs, which prevents from recovering a completely assembled genome. In the following section, we generalize the approach described above to allow for a more flexible notion of synteny in term of gapped-adjacencies, which addresses the above-mentioned problem.

### Generalization to gapped adjacencies

Before describing our new algorithm called GapAdj, which is a generalization of DirectAdj accounting for *α*-adjacencies for increasing values of *α*, we start by motivating our new approach.

**Figure 3:**
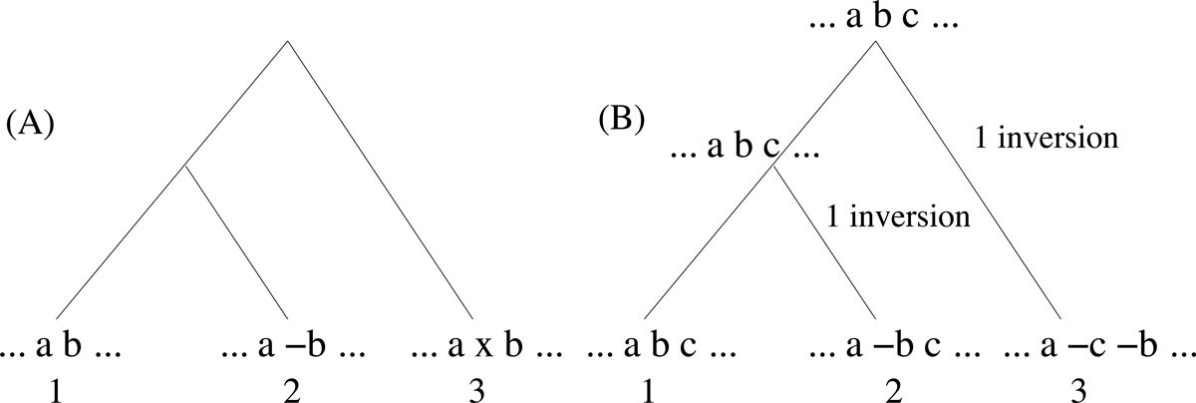
**A species tree for the set of species Γ = {1, 2, 3}, with two different genome assignments at leaves**. Example (B) depicts a most parsimonious inversion scenario leading to the observed genomes.

Many adjacencies in an ancestral genome are likely to be no longer present in some present-day genomes due to rearrangements and content-modifying operations, preventing from reconstructing large CARs. However, since small and local evolutionary events are more frequent than large and far-reaching operations [21], we can expect to reconnect neighboring CARs by considering gapped adjacencies. Consider for example the species tree (A) of Figure 3. As *a *and *b *are "neighboring" (close) genes in all three genomes, we expect the inferred ancestral genome at the root of the tree to have a CAR with genes *a *and *b*. However, as all (right) direct adjacencies of *a *are different (*b *in 1, -*b *in 2 and *x *in 3), none of these adjacencies would have a score attaining a reasonable minimum cost *τ *for the TSP, and *a *and *b *will end up in two different CARs with algorithm DirectAdj. However, as *b *(and also -*b*) is a 2 - *adjacency *of *a *in two extent genomes, and a 3 - *adjacency *of *a *in all three genomes, they end up in the same CAR if we consider 2 or 3-adjacencies (second or third iteration of GapAdj algorithm described in the next section). As another example, consider a "true" evolutionary scenario depicted in Figure 3.(B). Consider a threshold *τ *for TSP-*τ *corresponding to an adjacency being present in two of the three extent species. Then, as the only direct adjacency present at least twice in extant genomes is *bc*, DirectAdj leaves *a *and *bc *in two separate CARs. However, as *b *is *a *2-adjacency of a in species 1 and 2 (it is actually the only adjacency reaching the threshold up to *α *= 2), GapAdj would end up with a CAR containing the sequence *abc *after iteration *α *= 2.

**Algorithm Gapped-Adjacencies (GapAdj): **$\left(\sum ,S,v,\tau ,MAX_{\alpha }\right)$

Initialize the set *C *of CARs to $\sum_{v}$;

**For ***α *=1 to *MAX_α _***Do**

Step 1:

1. **For **each internal node *u *of *S *(bottom-up traversal) **Do**

2.    **For **each $g\in \sum_{u}$**Do**

3.       **For **each multiset *X *of possible adjacencies of *g *at *u ***Do**

4.             Compute *LeftAdj*(*g, α, S*|_*LA*(*g,α,G*(*u*)) = *X*_);

5.             Compute *RightAdj*(*g, α, S*|_*RA*(*g,α,G*(*u*)) = *X*_);

      **End For**

   **End For**

 **End For**

Step 2:

 Construct the graph *Q *with vertices being the genes of , and edges weighted according to computed *α*-adjacencies; By applying TSP-*τ *on *Q*, update the set *C *of CARs; Restrict  to the *α*-extremities of each CAR of *C*;

End For

Return (*C*);

#### Algorithm

The full GapAdj algorithm is given in Algorithm Gapped-Adjacencies. The output of GapAdj is the set of CARs *C *representing the ancestral genome at node *ν *of *S*. This set is first initialized to the set Σ_*ν *_of genes at *ν *(each gene being assigned to its own CAR). The algorithm proceeds by iterating the two-step procedure described in the section on direct adjacencies on increasing values of *α*, from 1 to a constant *MAX_α_*. Step 1 consists in computing *α*-adjacency scores. The dynamic programming algorithms detailed in [2] for computing the scores $LeftAdj\left(g,S\;{|}_{LA\left(g,1,G\left(u\right)\right)=X}\right)$ and $RightAdj\left(g,S\;{|}_{RA\left(g,1,G\left(u\right)\right)=X}\right)$of left and right adjacencies of a gene *g *with a multiset *X *at a node *u *of *S *are directly generalizable to account for *α*-adjacencies, i.e. to compute the scores $LeftAdj\left(g,S{|}_{LA\left(g,\alpha ,G\left(u\right)\right)=X}\right)$ and $RightAdj\left(g,S\;{|}_{RA\left(g,\alpha ,G\left(u\right)\right)=X}\right)$. As for Step 2, we proceed by constructing a complete undirected graph *Q *where vertices are the two extremities of each CAR, and edges are weighted according to *α*-adjacencies scores, computed at Step 1, of the two genes at the extremities of each CAR. A heaviest Hamiltonian cycle through *Q*, where edges with weight under a threshold *τ *are excluded, corresponds to an hypothetical ancestral genome characterized by a set of CARs *C_α _*with |*C_α_*| ≤| *C*_*α*-1_|. This instance of the TSP is solved using the Chained Lin-Khernigan heuristic implemented in the Concord package [22].

An important parameter of our algorithm is the cut-off value *τ *used to filter out less reliable adjacencies from the solution produced by the TSP algorithm. Based on the simulations that we have performed in [2], we choose a fixed threshold allowing for the best balance between error rate and number of CARs produced. The chosen threshold *τ *corresponds roughly to keeping an adjacency if and only if it is conserved in at least 70% of the tree branches. Another important parameter of our algorithm is the constant *MAX_α_*, corresponding to the maximum value of *α *to be considered, which affects both the running time, the final number of CARs and their accuracy. Clearly *MAX_α _*does not need to be more than the size of the longest chromosome of Γ, as no improvement can be achieved for larger values. Unless explicitly indicated, we use *MAX_α _*= 50, which produced good results in most of our simulations.

#### Complexity

As the complexity of Step 2 depends upon the considered heuristic for the TSP, we focus here on the complexity of Step 1. Denote by *mult *the highest multiplicity of a gene *g *at any node of *S*. It follows from the complexity result given in [2] (section 4.1) that each line 4. and 5. of algorithm GapAdj can be computed in time *O*(|*S*||Σ|*^mult^*). The For loop 3. multiplies this complexity by |Σ|, the same holds for the For loop 2., and the For loop 1. multiplies this complexity by |*S*|. It follows that Step 1 of algorithm GapAdj can be computed in time $O\left(Max_{\alpha }\times |S{|}^{2}\times |\sum {|}^{mult+2}\right)$.

## Results and discussion

**Figure 4:**
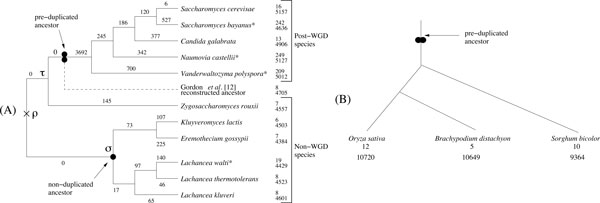
**(A) Evolution of the 11 yeast species recorded in the Yeast Gene Order Browser, as given by **[29]. The * indicates partially sequenced organisms. At leaves, the top number is the number of chromosomes, contigs or scaffolds. The bottom number is the number of genes, as reported in [18]. On each branch, the label is the number of gene losses, which is directly inferred from the gene content at leaves. The simple circle is the root of the monophyletic group of non-duplicated species, referred in the text by *σ*. (B) The phylogenetic tree for *Oryza sativa *(rice), *Brachypodium distachyon *(brachypodium) and *Sorghum bicolor *(sorghum). At leaves, the top number is the number of chromosomes. The bottom number is the number of markers used in the study of cereal genomes.

To evaluate the accuracy and running time of our approach, we first used data generated using simulated genome evolution. This allows us to dissect the impact of each aspect of the method and of the data on the accuracy of the reconstructed ancestor. Our simulations are based on the phylogenetic tree of yeast species shown in Figure 4 (A), which is ideal for this type of study as it contains a phylum affected by a whole-genome duplication and another that remains non-duplicated. Each of the simulation-based results reported in this section are averaged over 50 repetitions.

### Simulations with no WGD

In the absence of WGD events, the method that is most comparable to ours is the one of Ma *et al*. [5], implemented in a program called InferCAR. As this method does not support gene losses, we first restrict our simulations to a model with rearrangements only. In addition, as a first validation, we consider single chromosomal genomes, and inversions as the only rearrangement events.

We simulated data sets based on the yeast phylogenetic tree but excluding the portion affected by the WGD. The tree contains six non-duplicated species. The node of interest is the root *σ *of the monophyletic group of five species (indicated by a simple circle in Figure 4 (A)). A simulated genome of two hundred genes is placed at the root *ρ *of the tree, and a number *r *of inversions are randomly performed on each branch of the tree, where *r *is chosen randomly in the interval $\left[\frac{rmax}{2},rmax\right]$, for a given constant value *rmax*. While the theoretical time complexity of GapAdj is reasonable for genomes with thousands of genes, the unoptimized state of the implementation renders the execution of multiple simulations with larger genomes rather time consuming, hence the choice of an ancestral genome with only two hundred genes. However, intuitively one can see that similar results could be obtained with larger genomes if the number of rearrangements is increased proportionally. Notice that the maximum value *rmax *= 25 considered in our simulations leads to some of the leaf genomes being almost completely shuffled, as four or five branches separate them from the root, which lead to the creation of about 160 to 200 breakpoints. The length of inverted segments follows a geometric distribution with *p *= 0.5, resulting in a majority of short inversions, as previously suggested [21].

**Figure 5:**
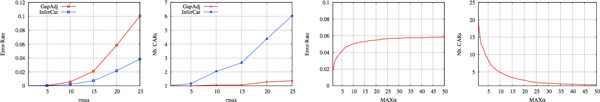
**Simulations for a tree without WGD, and a maximum of *rmax *inversions (x-axis on the two left diagrams) on each branch**. Red curves are the results of GapAdj and the blue ones those of InferCAR. From left to right, (1st): Error rate for the inferred ancestral genome; (2nd) Number of inferred CAR; (3d) Error rate and (4th) Number of CARs obtained by *GapAdj *. For these two diagrams *rmax *= 20 and values on the *x*-axis correspond to the parameter *MAX_α_*.

Figure 5 (two left diagrams) illustrates the two algorithms' error rates, computed as the fraction of inferred *α*-adjacencies (for 1 ≤ *α *≤ *MAX_α_*) that are not present as *α*-adjacencies in the true simulated ancestor at *σ*, while the right diagram illustrates the number of CARs obtained (on average) for that ancestor. Both algorithms show a high accuracy for adjacency prediction, as the error rate is always lower than 10%. Our GapAdj algorithm almost always recovers a complete genome (i.e. a single CAR), which is very rarely the case of InferCAR, which yields an average of 6 CARs for *rmax *= 25. However, this increase in CAR concatenation is obtained at the cost of a certain loss of precision.

Figure 5 (two right diagrams) illustrates the progression of the error rate and CAR number for increasing values of *α*. It provides a comparison with the initial algorithm DirectAdj [2] that only considers direct adjacencies (*α *= 1). From *α *=1 to *α *= 50, the number of CARs drops from 20 to a single chromosome, while the error rate is increased by less than 4%. This increase in error rate is expected, as CARs being joined together for *α *> 1 do not have strong support based on direct adjacencies and are necessarily more difficult to infer. Nonetheless, we note that the increase in error rate is relatively modest, compared to what would be expected if CARs were joined randomly, which would produce an increase of approximately 10% in error rate. These preliminary results are promising as the initial goal of obtaining a completely assembled genome while keeping a low error rate is attained in this case.

**Figure 6:**
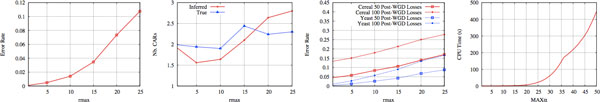
**From left to right, (1st) Error rate and (2nd) Number of CARs obtained by *GapAdj *on simulations following a model accounting for multichromosomal genomes evolving through gene losses, and a maximum of *rmax *(x-axis) inversions and inter-chromosomal rearrangements per branch of the tree**. (3d) Error rate obtained by *GapAdj *on simulations performed according to the cereal tree (Figure 4(B)) and the subtree of yeast rooted at *τ *(Figure 4(B)). The model accounts for inversions, inter-chromosomal rearrangements, gene losses and one WGD. The two red (resp. blue) curves correspond to the results for cereal (resp. yeast) by performing 50 and 100 losses just following the WGD. (4th) Running time of *GapAdj *for one data set following the "cereal 50" model, and with *rmax*=20.

We then consider an extended model of evolution for multichromosomal genomes that evolve through inversions, inter-chromosomal rearrangements (translocations, fusions, fissions) and gene losses. Based on the same six-leaf species tree described above, we simulate data sets starting with a 2-chromosome, 200-gene genome at the root *ρ *of the tree. Each gene loss event involves a single gene chosen randomly in the genome. The number of gene losses on each branch is proportional to that observed in actual yeast genomes, while the proportion of each type of rearrangement operation is chosen to be similar to that reported for *S*. *cerevisiae *in [18]: (Inv : Trans : Fus+Fiss) = (5 : 4 : 1). The results given in Figure 6 (two leftmost diagrams) reflect the difference in gapped-adjacencies and number of chromosomes between the real and predicted genome at node *σ*. Notice that chromosomal fusions and fissions may occur on the branch from *ρ *to *σ*, so the true number of chromosomes depicted in the second diagram of Figure 6 is not always 2. Interestingly, the curve for inferred CARs roughly follows the curve for true CARs. In addition, the error rate remains lower than 12% in all cases.

### Simulations with WGD

For simulations with WGD, we used two trees: one being the subtree of yeast (Figure 4 (A)) rooted at *τ*, and another (Figure 4 (B)) corresponding to the evolution of three cereals (rice, brachypodium and sorghum), that we will study thereafter. We simulate data sets starting with a pre-duplication 2-chromosome, 200-gene genome at the root of the tree and performing a number of gene losses and a maximum *rmax *of rearrangements on each branch. As WGD events are usually followed by extensive losses, we perform 50 or 100 random losses between the duplication and first speciation event, followed by 5 random losses on each branch of the tree. As for the rate of various rearrangements, we use the same as before. Error rates are given in Figure 6 (third diagram). The number of CARs produced by the algorithm typically slightly overshoots the correct number, varying from 2 to 4. Note that the losses that occurred immediately after the duplication event result in many false adjacencies inferred, as depicted by the difference in error rate between simulations with only 50 post-duplication losses and those with 100. Since those are ancient events, their effects are seen on many or all of the leaf gene orders, preventing us from inferring the right order in areas surrounding the lost genes in the ancestor. Interestingly, the fact that an outgroup predating the WGD is available for yeast allows to circumvent this problem as adjacencies can be grasped from this genome not affected by losses, which explains the better results obtained for yeast. Figure 6 (last diagram) shows the running time of our algorithm for *rmax *= 20, as a function of *MAX_α_*. Although the running time increases cubically with *MAX_α_*, it remains quite manageable. In the absence of the WGD, the running time is significantly smaller, as it remains under 2 seconds even for *MAX_α _*= 50.

### Study of yeast genome evolution

**Figure 7:**
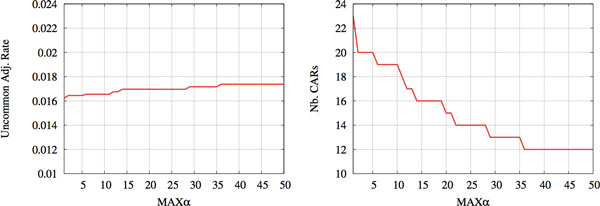
**(Left) Fraction of adjacencies in disagreement between the pre-duplicated yeast ancestor inferred by *GapAdj *and that inferred by Gordon et al**. in [18]. (Right) Number of CARs inferred with *GapAdj *algorithm.

We applied our method to the full yeast species tree (Figure 4 (A)) with the gene data sets of the *Yeast Gene Order Browser *[18], to infer the pre-duplicated ancestral genome of *Saccharomyces cerevisiae*. Both this dataset and the cereals dataset had been curated by their authors to remove genes whose duplication is likely to come from events other than WGDs. We then compared our predicted ancestor with the 8-chromosome genome manually inferred by Gordon *et al*. [18]. Figure 7 (left) gives the fraction of *α*-adjacencies that we infer but are in contradiction with the genome inferred by Gordon *et al*. For all tested values of *α*, this fraction remains below 2%. Importantly, considering gapped adjacencies in addition to direct adjacencies allows to reduce the number of CARs from 23 to 12, which is significantly closer to the number of ancestral chromosomes predicted by Gordon *et al*. Among the 11 additional inferred 1-adjacencies, 7 are shared with the ancestor of Gordon *et al*.

### Study of cereal genome evolution

We now focus on three of the four completely sequenced cereal crop genomes studied by Murat *et al*. [23], namely rice (*Oryza sativa*), sorghum (*Sorghum bicolor*) and brachypodium (*Brachypodium distachyon*). As demonstrated by various studies, these species have evolved following a whole genome duplication that has occurred about 60 million years ago (see Figure 4.(B)). Maize, the fourth species considered in [23] was excluded here to avoid noise due to an additional maize-specific WGD and ensuing massive gene loss. We used the sets of markers (10,720 from rice, 10,649 from brachypodium, and 9,364 from sorghum) and the homology relationships provided by Murat *et al*., and the orders for these markers from [24-26].

**Figure 8:**
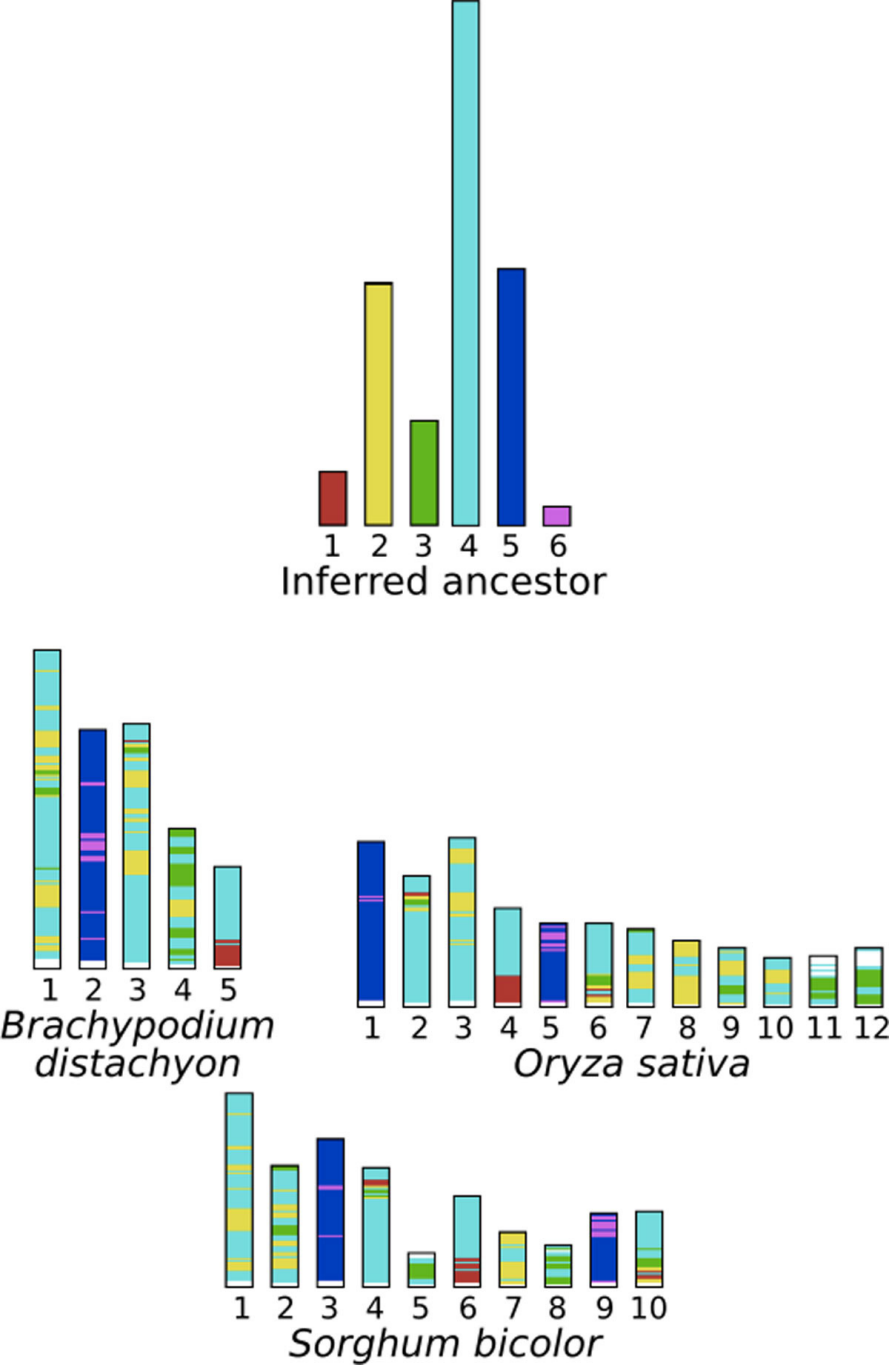
**Syntenic regions of three cereal species karyotype with respect to their ancestor inferred using our *GapAdj *algorithm**.

Figure 8 shows the predicted pre-duplication genome and its extant descendants. Syntenic regions (homologous sets of genes with conserved order) are painted using the Cinteny web server [27]. Running GapAdj with a maximum value of *α *(up to the size of the largest chromosome which is about 3500), we end up with a set of 6 CARs (plain bars in Figure 8), which is one more chromosome than that inferred by Murat *et al*. [23]. Looking carefully at the obtained results, we can see that the ancestral CARs 5 and 6 are clustered (and shuffled) into a single chromosome in Brachypodium (chromosome 2), and in two chromosomes in rice and sorghum (chromosomes 1 and 5 in the rice, and 3 and 9 in sorghum). Moreover there is no other segment of the CARs 5 and 6 in any other extant chromosome. This observation suggests that these two CARs should be concatenated into a single and complete chromosome. This would be consistent with the results reported by Murat *et al*. [23], who infer that a single pre-duplicated chromosome *C *is the ancestor of the same chromosome in Brachypodium (2) and the same two chromosomes in rice (1 and 5) and sorghum (3 and 9). The reason our algorithm did not concatenate them is probably that the genes at both extremities of the ancestral CAR 5 are in two different chromosomes in rice and sorghum. This suggests a future extension of our algorithm that would consider the *α*-extremities of each current CAR for subsequent concatenations.

Comparing our observations with Murat *et al*., we notice a number of striking similarities. In particular, one of the main discovery of the paper [23] is that some chromosomes have evolved following a particular evolutionary event, called nested fusion, resulting in the insertion of one chromosome inside another (non-telomeric fusion). Indeed, chromosome 2 of Brachypodium is explained in [23] as resulting from a nested chromosome fusion of the two copies of the chromosome *C *(introduced in the previous paragraph), that has occurred after the speciation leading to the Brachypodium lineage. Interestingly this nested fusion is clear in our results, as our chromosome painting is in agreement with chromosome 2 of Brachypodium being the result of an insertion of the ancestors of rice chr. 5 in the middle of the ancestor of rice chr. 1.

## Conclusions

Any method for ancestral genome inference is debatable by nature, as it should be based on a model of evolution that is set *a priori*, even though we have no direct access to the history of genomes. Moreover, as real ancestors are not known, any validation method is open to criticism, and there is no direct way of evaluating one solution compared to another. Based on the first observation, we opted for a synteny-based method that is based as much as possible on the observed data sets, without the need for explicitly defining the rearrangement events acting on these genomes. It is the first synteny-based method that fully capitalizes on the observed adjacencies in present day genomes in relation with their phylogenetic organization. It is flexible enough to apply to genomes that have evolved through WGD events, rearrangements and gene insertions and losses. Based on the second observation, we first opted in [2] for a conservative approach concatenating two ancestral genes *g *and *h *only if the direct adjacency (*g, h*) is observed in a large fraction of extant genomes and sufficiently supported by the phylogeny. The result was an algorithm with high accuracy for adjacency prediction, but with the counterpart being a high number of CARs. Our generalization to gapped adjacencies while maintaining a conservative strategy for each gap size has led to a reasonable compromise between accuracy in adjacency and karyotype reconstruction.

A clear limitation of most empirical and analytical approaches considering evolution by WGD [9,10,15-19], including our method, is to ignore all other sources of gene duplication. This is not to say that single-gene duplications are assumed not to happen during evolution, but rather that a preprocessing of the genomes eliminating all undesired gene copies is done preliminary to applying the developed methodology. As discussed in Byrne et al. [28] in the case of yeast and Murat et al. [23] in the case of cereals, gene families that have undergone recent expansions via smaller tandem or segmental duplications can typically be identified via a phylogenetic analysis or other homology based approaches. However, in general identifying the true orthologous and paralogous gene copies is not an easy problem. Generalizing our approach to account for local gene duplication would therefore be an interesting future work, although the complexity of the optimization problem is expected to be considerably increased, as well as the size of the solution space (the number of most parsimonious evolutionary histories).

## Competing interests

The authors declare that they have no competing interests.
